# Quaternary climate instability is correlated with patterns of population genetic variability in *Bombus huntii*

**DOI:** 10.1002/ece3.4294

**Published:** 2018-07-13

**Authors:** Jonathan B. Koch, Rémy Vandame, Jorge Mérida‐Rivas, Philippe Sagot, James Strange

**Affiliations:** ^1^ Department of Biology Utah State University Logan Utah; ^2^ Pollinating Insects Research Unit USDA‐ARS‐PWA Logan Utah; ^3^ Departamento Agricultura Sociedad y Ambiente El Colegio de la Frontera Sur San Cristóbal de Las Casas Chiapas Mexico

**Keywords:** *Bombus huntii*, climate change, isolation by distance, isolation by resistance, last glacial maximum, latitude gradient, pollinator

## Abstract

Climate oscillations have left a significant impact on the patterns of genetic diversity observed in numerous taxa. In this study, we examine the effect of Quaternary climate instability on population genetic variability of a bumble bee pollinator species, *Bombus huntii* in western North America. Pleistocene and contemporary *B. huntii* habitat suitability (HS) was estimated with an environmental niche model (ENM) by associating 1,035 locality records with 10 bioclimatic variables. To estimate genetic variability, we genotyped 380 individuals from 33 localities at 13 microsatellite loci. Bayesian inference was used to examine population structure with and without a priori specification of geographic locality. We compared isolation by distance (IBD) and isolation by resistance (IBR) models to examine population differentiation within and among the Bayesian inferred genetic clusters. Furthermore, we tested for the effect of environmental niche stability (ENS) on population genetic diversity with linear regression. As predicted, high‐latitude *B. huntii* habitats exhibit low ENS when compared to low‐latitude habitats. Two major genetic clusters of *B. huntii* inhabit western North America: (a) a north genetic cluster predominantly distributed north of 28°N and (b) a south genetic cluster distributed south of 28°N. In the south genetic cluser, both IBD and IBR models are significant. However, in the north genetic cluster, IBD is significant but not IBR. Furthermore, the IBR models suggest that low‐latitude montane populations are surrounded by habitat with low HS, possibly limiting dispersal, and ultimately gene flow between populations. Finally, we detected high genetic diversity across populations in regions that have been climatically unstable since the last glacial maximum (LGM), and low genetic diversity across populations in regions that have been climatically stable since the LGM. Understanding how species have responded to climate change has the potential to inform management and conservation decisions of both ecological and economic concerns.

## 1 INTRODUCTION

Geographic instability of ecosystems due to Quaternary climate change has left a lasting imprint on the compostion and diversity of populations and species across the planet (Hewitt, 1996, 2000). Specifically, climate oscillations since the Pleistocene have facilitated both population divergence and speciation through isolation and recolonization of suitable habitats (Callahan et al., 2013; Carvalho et al., 2017; Galbreath, Hafner, Zamudio, & Agnew, 2010; Gutiérrez‐Rodríguez, Barbosa, & Martínez‐Solano, 2016; Knowles, 2000). A decrease in the geographic spread of suitable habitat over time may lead to a population range contraction, cascading toward a population bottleneck, genetic drift, and a possible loss of genetic diversity (Pauls et al., 2013). Alternatively, the geographic expansion of suitable habitat over time may facilitate a population expansion, which may also lead to a loss of genetic diversity due to founder effect, as the establishing population is typically made up of a small number of colonizing individuals (Pauls et al., 2013). However, colonization into new suitable habitat may also attract individuals from a diverse pool of populations and result in an increase in population genetic admixture (Ortego, Gugger, & Sork, 2015). Understanding how biodiversity responds to environmental change has the potential to inform effective management decisions for species of ecological and economic concern.

The availability of microsatellite markers and environmental niche modeling techniques provides the opportunity to examine the effects of late Pleistocene climate variability on population genetic variability (Callahan et al., 2013; Gutiérrez‐Rodríguez et al., 2016; López‐Uribe, Zamudio, Cardoso, & Danforth, 2014). There is converging evidence that species have responded to climate oscillations through either geographic expansion or contraction, depending on their associated life history traits and ecological demands (Callahan et al., 2013; Galbreath et al., 2010; López‐Uribe et al., 2014). Small rodents with limited dispersal capacity due to narrow bioclimatic niches exhibit strong population divergence as suggested by highly conserved gene regions during the Pleistocene (Galbreath, Hafner, & Zamudio, 2009). However, animals with great dispersal ability and broad bioclimatic niches have received less attention until recently (Françoso et al., 2016; López‐Uribe et al., 2014). Furthermore, many studies have examined species with strong bioclimatic specialization (e.g., montane/alpine specialist), with few studies examining species that may exhibit a degree of adaptation to regional climate variability. Correlating climate variability with neutral population genetic variability has the potential to build an inference on the role of global environmental change as a driver of population isolation and genetic drift.

Bumble bees (Hymenoptera: Apidae, *Bombus*) are an appropriate organismal model for studying the effects of climate oscillations on contemporary bioclimatic niches, range dynamics, and population‐level divergence during the Quaternary. They are large‐bodied insects and have the capacity to disperse over several kilometers in search of food, nesting sites, and hibernacula (Jha, 2015; Lozier, Strange, & Koch, 2013; Woodard et al., 2015). Furthermore, bumble bees are densely covered in setae and can fly at low temperatures by warming their wing muscles prior to flight (Heinrich & Esch, 1994). They are dependent on pollen and nectar from flowers to feed developing brood. Thus, dispersal and colonization during the Pleistocene have likely been affected by changes in the distribution of food plants. In western North America, several bumble bee species are associated with temperate, alpine environments that can act as sky islands (Heald, 1951; Lozier et al., 2013). These sky islands and adjacent valleys may have served as refugia for bumble bees during climate oscillations where they may have tracked their preferred habitat across different mountain provinces (Galbreath et al., 2009).

**Figure 1:**
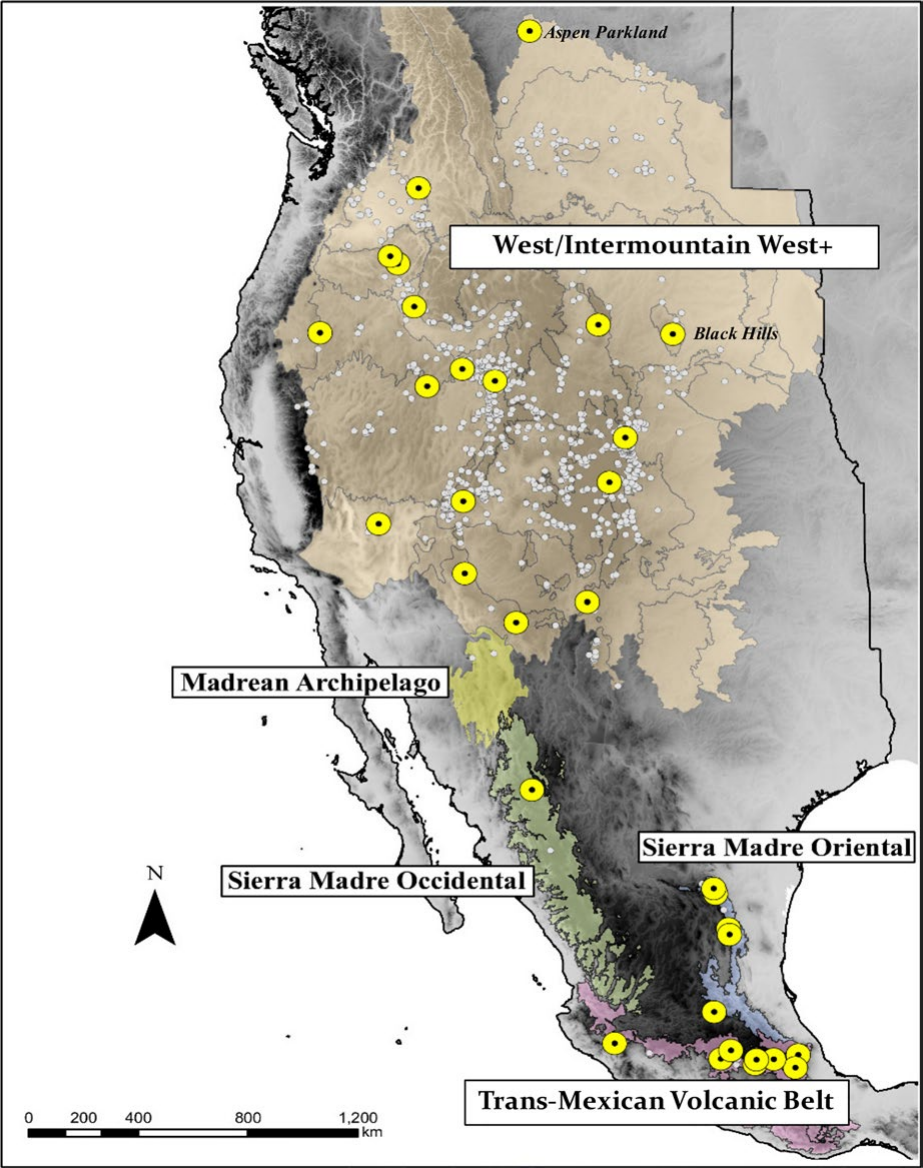
Natural history (NH) and current survey records of *Bombus huntii* throughout western North America. Current survey records = yellow point enclosing black point, NH record = white point. Except for “West/Intermountain West+,” all ecoregions presented here are adapted from Olsn et al. (2001). The gross delineation of the “West/Intermountain West+” on this map is modified from maps developed for waterbirds by Ivey and Herzinger (2006). Locality data are presented in decimal latitude and longitude with the WGS1984 coordinate system and the North American Albers Equal Area Conic geographic projection

In this study, we examine patterns of contemporary population genetic variability of the Hunt bumble bee, *Bombus huntii* Greene, 1860 across its geographic range. The latitudinal distribution of *B. huntii* extends from the southern edge of Canada, south throughout the Intermountain West, and Front Range of the Colorado Rocky Mountains to the Trans‐Mexican Volcanic Belt province in southern Mexico (Koch, Strange, & Williams, 2012; Labougle, 1990; Thorp, Horning, & Dunning, 1983). The longitudinal range of the species is primarily bound by the crest of the Sierra Nevada and Cascade Mountains in the west, and the Black Hills of South Dakota in the east (Figure 1). Although populations of *B. huntii* have been found east of the montane environment of South Dakota, they have been documented in low abundance relative to surveys of bumble bee communities in Nevada, Utah, Colorado, Montana (Dolan et al., 2017; Koch et al., 2015). Finally, *B. huntii* and its sibling species *B. vosnesenskii* diverged from their most recent common ancestor by the early Pliocene (~5 mya) (Cameron, Hines, & Williams, 2007; Hines, 2008). Thus, contemporary population genetic diversity of *B. huntii* can be investigated using climate scenarios estimated for the Pleistocene.

The primary objective of this study was to examine the role of Quaternary climate oscillations on population genetic variability across *B. huntii* populations. First, we construct environmental niche models (ENMs) based on contemporary occurrence records and bioclimatic variables. Then, operating under the principle of niche conservatism (Peterson, Sober, & Sanchez‐Cordero, 1999), we generated a habitat suitability (HS) map of *B. huntii* during the last glacial maximum (LGM) (~ca. 22,000 years before present, ybp). We predict that high‐latitude populations will exhibit greater niche instability compared to low‐latitude populations since the LGM due to the presence of the glaciated regions in northern latitudes (Callahan et al., 2013). Second, we used Bayesian inference to test for differences in genetic structure across contemporary *B. huntii* populations. We predict that high‐latitude populations will exhibit high genetic diversity as they are distributed across a broad elevation gradient and will be able to disperse throughout the environment and maintain gene flow, whereas low‐latitude populations are restricted to high‐elevation environments which likely impede gene flow (Koch, Looney, Sheppard, & Strange, 2017). Third, we test for the effect of geographic distance and environmental resistance on contemporary patterns of population differentiation across *B. huntii* with isolation by distance (IBD) and isolation by resistance models (IBR), respectively. Bumble bees are sensitive to environmental variation; thus, we predict that IBR will be a better predictor than IBD for examining population differentiation (Koch et al., 2017; Lozier et al., 2013). The goal of examining both IBR and IBD is to determine whether *B. huntii* genetic diversity estimates are best predicted by HS or geographic distance, respectively. Finally, we combined the contemporary and LGM HS maps to estimate an environmental niche stability (ENS) map to test the hypothesis that climate instability predicts contemporary genetic diversity across *B. huntii* populations. We predict that patterns of genetic diversity can be explained by ENS patterns, with stable areas (low‐latitude regions) associated with low genetic diversity and unstable areas (high‐latitude regions) associated with high genetic diversity (Callahan et al., 2013).

## 2 MATERIALS AND METHODS

### 2.1 Quaternary environmental niche modeling

To estimate the geographic distribution of HS of *B. huntii* throughout its endemic range at present and during the Pleistocene, ENMs were constructed under the principle of maximum entropy with MAXENT v3.3.1 (Phillips, Dudík, & Schapire, 2004). The software program MAXENT uses presence‐only georeferenced locality records and random background points sampled from the study extent to estimate the distribution of the species that is closest to uniform (=maximum entropy) under the suite of independent variables (i.e., bioclimatic variables) supplied to the model (Elith et al., 2011). Georeferenced distribution records were queried in the Global Biodiversity Information Facility database (http://gbif.org) and filtered for unique spatial coordinates (Figure 1) (Supporting Information Appendix S1). Additional records from a contemporary survey of Mexican bumble bees were included in the final set of georeferenced localities of *B. huntii* and are provided in Table 1. Specimen occurrence records that do not agree with historic and contemporary range maps of *B. huntii* were filtered out of the final dataset (Koch et al., 2012; Thorp et al., 1983; Williams, Thorp, Richardson, & Colla, 2014).

**Table 1:** Location description, sample size (*N*), and colony assignment of *Bombus huntii* in this study. Region assignment is inferred using a Bayesian genetic cluster assignment test with 11 microsatellite markers in STRUCTURE v 2.3.4 (Pritchard et al., 2000). Assignment of individuals to a colony is made with the program COLONY 2.0 (Jones & Wang, 2010)

Population	Population code	Region	Latitude	Longitude	*N*	No. of colonies
Ada	ADA	North	43.71	−116.30	10	10
Almoloya de Juarez	ALM	South	19.25	−99.87	11	11
Amecameca	AME	South	19.10	−98.68	30	26
Apache	APA	North	33.80	−109.15	14	14
Artega‐Galena	ARG	South	25.27	−100.43	32	31
Ayahualulco	AYA	South	19.46	−97.20	10	10
Baker	BAK	North	45.01	−117.58	10	10
Black Hills	BLH	North	43.98	−103.75	14	14
Box Elder	BOE	North	41.96	−113.45	9	7
Cache	CAC	North	41.74	−111.83	17	16
Chaffee	CHA	North	38.84	−105.99	5	5
Ciudad Guerrero	CIG	North	28.20	−107.60	12	12
Ciudad Serdan	CIS	South	19.00	−97.30	9	8
Clark	CLA	North	36.34	−115.65	26	25
Contla de Juan Camatzi	CJC	South	19.29	−98.05	4	4
Edmonton	EDM	North	53.53	−113.50	4	4
Elko	ELK	North	41.18	−114.86	12	12
Flagstaff	FLA	North	35.20	−111.63	12	12
Garfield	GAR	North	37.59	−112.26	14	13
General Zaragoza‐Miquiuana	GZM	South	23.70	−99.83	20	19
Ixtapaluca	IXT	South	19.26	−98.64	20	20
Jiquipilco	JIQ	South	19.57	−99.54	10	9
Lake	LAK	North	42.18	−120.35	17	17
Spokane	SPO	North	47.62	−117.51	23	23
Torrance	TOR	North	34.77	−106.33	4	4
Washakie	WAS	North	44.07	−107.38	15	14
Total					364	350
Average					14.16	13.6
Standard error					1.52	1.44

Occurrence records were aggregated with 18 spatially explicit bioclimatic variables representing contemporary conditions (1950–2000) from the WorldClim v1.4 Bioclim dataset (2.5 arc minutes) (Hijmans, Cameron, Parra, Jones, & Jarvis, 2005). To reduce model complexity, we examined the relationship between the 18 continuous bioclimatic variables with a pairwise Pearson correlation coefficient (*r*) test. From each pairwise correlation coefficient estimate, we retained only one variable for the final model if *r *≥* *0.75. Rather than randomly select a variable for the analyses, we chose to retain the variables that reflect annual and seasonal trends in precipitation and temperature as bumble bees are primarily active during the summer months, but also need to survive winter hibernation (Lozier et al., 2013). ENMs in MAXENT were constructed with default parameters to generate a logistic output (a measure of relative HS), averaged over 100 replicates with a subsampling scheme to evaluate model performance (75% train, 25% test). The *B. huntii* ENMs were evaluated in MAXENT using the area under curve (AUC) statistic and a permutation of variable importance. AUC values closer to 0.5 (random) suggest poor predictive performance, whereas values closer to 1 (nonrandom) suggests high predictive performance. Permutation tests of variable performance employed within the MAXENT software platform used the training points to assess the relative contribution of each variable to the final averaged model in the context of the AUC statistic. A significant drop in the AUC statistic after a bioclimatic variable is removed suggests that the variable significantly contributes to the estimation of HS. Locality data are presented in decimal latitude and longitude with the WGS1984 coordinate system and the North American Albers Equal Area Conic geographic projection. Geographic visualization of the ENMs was made with ArcGIS v10.1 (ESRI, CA).

Operating under the principle of niche conservatism (Peterson et al., 1999), we predicted the distribution of *B. huntii* during the LGM (~22,000 ybp) using the constraints of the contemporary bioclimatic associations of the *B. huntii* ENM mapped to paleoclimate data available within the WorldClim database (Hijmans et al., 2005). We used the CCSM4 fully coupled global climate model to estimate the *B. huntii* LGM environmental niche map. To identify potential climate refugia over the Quaternary, we added contemporary and LGM HS maps together to produce a ENS map. The raster output of the ENS map was standardized by dividing the calculated values by the raster's maximum value, producing values ranging from 0 (low niche stability) to 1 (high niche stability).

### 2.2 Contemporary taxon sampling

Female worker *B. huntii* were collected across 33 sampling sites throughout North America from 2008 to 2015 (Figure 1). This sampling regime captured a major portion of the species’ range in western North America (Labougle, 1990; Thorp et al., 1983). Bumble bees were sampled with a diversity of methods including sweep netting, colored bee bowls, and blue vane traps. Given that the aim of our study was to examine population genetic diversity and structure of wild *B. huntii*, we elected to pool certain sampling sites together if they were <9 km from each other. This decision was made based on known biological properties of dispersal in bumble bees, in that workers of the sister species, *B. vosnesenskii*, are estimated to disperse approximately 9 km from her nest (Jha & Kremen, 2013). We used this distance threshold as there are no current data on the landscape genetics of wild *B. huntii*. Furthermore, barriers to gene flow have been found to be influenced by both land‐use change and bioclimatic variability in *B. vosnesenskii* and *B. bifarius* (Jha & Kremen, 2013; Lozier et al., 2013). Thus, limiting the distance for pooling sites insures that we did not arbitrarily pool populations together and potentially alter genetic variability estimates.

### 2.3 DNA extraction and microsatellite genotyping

Within each of the 33 pooled sampling sites, an average of 12.25 (±1.29 *SE*) female bees were collected (*n *=* *380). DNA was extracted from the mid‐leg of each bumble bee using a modified‐Chelex100^™^ protocol described in Strange, Knoblett, and Griswold (2009) and screened at 13 microsatellite loci: B124, BTERN01, BTERN02, BT28, BT10, BT30, B96, BTMS0081, BTMS0066, BTMS0062, BL13, BTMS0044, and BTMS0059 (Estoup, Scholl, Pouvreau, & Solignac, 1995; Estoup, Solignac, Cornuet, Goudet, & Scholl, 1996; Reber Funk, Schmid‐Hempel, & Schmid‐Hempel, 2006; Stolle et al., 2009). Multiplex polymerase chain reactions (PCR) were performed in final volumes of 10 μl, containing approximately 1 μl of extracted DNA, 1× Promega (Madison, WI) reaction buffer, 0.6 mM dNTP mixture, 0.2–0.4 μM primer, 0.001 mg bovine serum albumin, and 0.4 units Taq polymerase (Promega, Madison, WI). The MgCl_2_ concentration was adjusted to 1.4 mM. The PCR conditions for both multiplex reactions were one cycle of 95°C for 3:30 min, 30 cycles of 95°C for 30 s, annealing temperature 55/58°C for 1:15 min, 72°C for 45 s, and a final extension period of 15 min at 72°C. The DNA amplifications were performed with fluorescent 5′‐end dye‐labeled primers (6‐FAM, NED, VIC, or PET) and separated on an Applied Biosystems 3730xl automatic sequencer at the Center for Integrated Biology at Utah State University. The allele sizes were scored manually using GENEIOUS v8 (Kearse et al., 2012). Only samples with ≥7 loci scored per individual were included in analyses.

### 2.4 Hardy–Weinberg equilibirium, linkage disequilibirium, and colony assignment

The probability of null alleles was estimated with the software program MICRO‐CHECKER (Van Oosterhout, Hutchinson, Wills, & Shipley, 2004). Deviations from Hardy–Weinberg equilibrium (HWE) and linkage disequilibrium (LD) across populations and loci were tested with the web‐based software program GENEPOP v4.0.10 (Raymond & Rousset, 1995). Sequential Bonferroni corrections were applied to the HWE and LD *p*‐values estimates to minimize type I errors associated with multiple comparisons for both populations and loci (Rice, 1989). We considered the Bonferroni correction test significant at *p *≤* *0.05.

As bumble bees are generally monandrous (Estoup et al., 1995), primitively eusocial, and live in annual colonies, it is possible to capture sibling female workers in the wild. To avoid pseudo‐replication within sampling locations, full‐siblings were first assigned to colonies with COLONY v2.0 (Jones & Wang, 2010). In the colony‐assignment exercise, we set the mistyping error rate to 0.05, based on error rates documented in previous studies (Lozier, Strange, Stewart, & Cameron, 2011), and the sex‐determination system to “haplodiploid.” If full‐siblings were detected in the colony‐assignment tests (≥95% genotype similarity), we selected only one representative from each family using a coin toss. Postanalysis mistyping error estimates are described in Supporting Information Appendix S2.

### 2.5 Population genetic structure and landscape genetics

Population genetic structure was examined with an individual assignment Bayesian clustering method in the software program STRUCTURE v 2.3.4 (Pritchard, Stephens, & Donnelly, 2000). We elected to use the admixture model in STRUCTURE to assign genotypes, which assumes that individuals comprise *K* unknown clusters (i.e., population), to which an individual can be assigned based on their genotype without a priori delineation of populations. We set the model to run with 20,000 burn‐in steps and 100,000 samples, with 10 iterations for each *K*, where *K* ranged from 1 to 10. To determine the optimal *K*, the distributions of the probability of the data *LnP*(*K*) and Δ*K* (as described by Earl & vonHoldt, 2012; Evanno, Regnaut, & Goudet, 2005) were examined with the web‐based software program STRUCTURE HARVESTER 0.6.94 (Earl & vonHoldt, 2012). To account for multimodality associated with individual STRUCTURE runs, we averaged an individual's admixture proportions over the 10 replicates for the best *K* using the full search algorithm in CLUMPP v1.1.2 (Jakobsson & Rosenberg, 2007). We visualized populationassignment by averaging *K*‐assignments within each population with pie charts in ArcGIS v10.1 (ESRI, 2012).

To compliment our STRUCTURE analysis, we also used the program GENELAND v4.05 (*Geneland* R library) to infer poplation structure (Guillot et al.*,* 2005). Unlike the STRUCTURE analysis, we included an examination of the effect of geographic location on individual assignment to cluster *K* in GENELAND. Locality data were transformed from decimal latitude and longitude to UTM with the *PBSmapping* R library. We set the GENELAND population assignment model to run with the following parameters: 1,000,000 Monte Carlo Markov Chain (MCMC) iterations with every 1,000th iteration saved; our maximum Poisson at 100; spatial coordinate uncertainty at 1; maximum number of nuclei in the Poisson–Voronoi tessellation to 350; and applied the null allele, uncorrelated frequencing, and spatial models. Ten independent runs were performed for *K* values from one to ten. To determine the best fit of the microsatellite genotypes, we chose the independent run with highest log likelihood values following a burnin of 100, 200, 300, 400, and 500. In addition to examining log likelihood values, we assessed the MCMC plot to determine the stationarity of the model along the chain, as well as the number of populations inferred from an indendepent run.

We estimated pairwise population differentiation with multilocus *F*_ST_ (Weir & Cockerham, 1984), which were transformed to *F*’_ST_ with GENALEX v6.5 Peakall and Smouse (2012). We treated localities as populations and tested for IBD with Mantel tests by calculating pairwise geographic distances between populations and using the associated pairwise *F*’_ST_. Pairwise geographic distance used for IBD analyses was calculated using the associated decimal latitude and longitude coordinates (Geographic Projection: WGS 1984). We first examined pairwise genetic differentiation by geographic distance by incorporating all the sampling sites genotyped in a single test of IBD. However, we also partitioned the dataset based on the STRUCTURE results to test for the effect of geographic distance on pairwise *F*’_ST_ within a defined genetic cluster.

In addition to testing for a correlation in IBD, we determined the capacity for contemporary HS to predict observed patterns of genetic structure (IBR) (Lozier et al., 2013; McRae, 2006; McRae, Dickson, Keitt, & Shah, 2008). We applied circuit theory, implemented in CIRCUITSCAPE v3.5.8 to estimate resistance distance matrices between all pairs of sampling sites (Shah & McRae, 2008). CIRCUITSCAPE uses electrical circuit theory to estimated resistance distance between two points to calculate the likelihood of potential gene flow, integrating over all possible pathways of dispersal. We used the logistic output from the contemporary HS raster as an input to estimate resistance within the landscape inhabited by *B. huntii*. We implemented CIRCUITSCAPE in pairwise mode with an eight‐neighbor cell connection scheme using average resistances and set the raster conductance so that values closer 1 (high HS) would reduce resistance and values closer to 0 (low HS) would increase resistance. Significance of geographic distance or environmental resistance between paired populations was tested with Mantel tests with *ecodist* R library (Goslee & Urban, 2007).

Both IBD and IBR patterns are hypothesized to capture the relationship between gene flow and genetic drift in the context of geographic and environmental distribution of populations. Our inference on the best predictor (geographic in IBD or environmental in IBR) was based on the Mantel correlation coefficient (*r*) fit to the genetic data. Higher *r* values suggest a better fit of the IBD or IBR variable to the pairwise *F*’_ST_ estimates, granted that they are significant at an alpha level of *p* ≤ 0.05.

### 2.6 Population genetic diversity

We estimated genetic diversity with two different metrics: (a) effective allelic diversity (*AD*) and (b) expected heterozygosity (*H*_e_) using Nei's genic diversity metric. Given our unequal sampling across field sites, we used rarefaction to estimate genetic diversity by standardizing our estimates to four gene copies per population (Kalinowski, 2005). We tested for the effect of ENS (based on the ENS map) on average *AD* and *H*_e_ with simple linear regression. Estimates of genetic diversity were made with the *gstudio* library available on the R statistical computing platform (Dyer, 2014).

### 2.7 Environmental niche comparisons

We used two sample Wilcoxon rank‐sum tests to test for significant bioclimatic differences between the two Bayesian‐infered genetic clusters (i.e., North vs. South cluster). Unless otherwise indicated, all statistical tests conducted in our study were executed with the R statistical computing platform with the appropriate statistics libraries (R Core Development Team, 2005).

## 3 RESULTS

### 3.1 Quaternary environmental niche modeling

**Figure 2:**
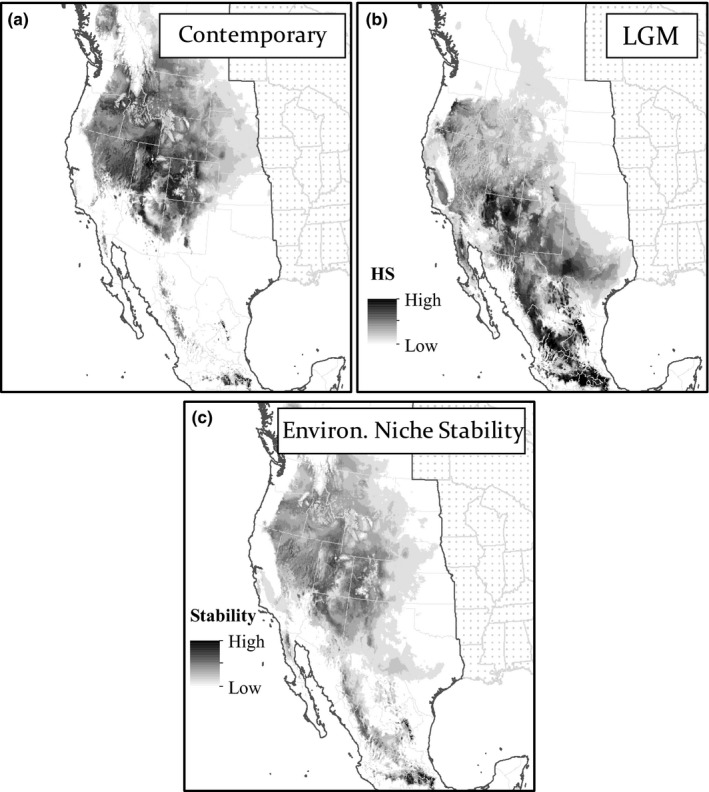
Estimate of *Bombus huntii* habitat suitability (HS) based on an environmental niche model (ENM) constructed with 10 bioclimatic variables (1950–2000). (a) Contemporary *B. huntii* HS map; (b) last glacial maximum *B. huntii* HS map, and (c) HS values across the contemporary and LGM time periods were added together to create an environmental niche stability map. Maps are presented in the North American Albers Equal Area Conic geographic projection. HS values closer to 1 suggest high HS (Black), whereas HS values closer to 0 suggest low HS (White). HS < 0.10 is in white

The 18 available bioclimatic variables were reduced to 10 bioclimatic variables after the assessment of colinearity and were incorporated into the final ENMs. The bioclimatic variables used to estimate HS included annual mean temperature (BIO 1), annual precipitation (BIO 12), precipitation seasonality (BIO 15), precipitation of warmest quarter (BIO 18), precipitation of coldest quarter (BIO 19), mean diurnal range (BIO 2), max temperature of warmest month (BIO 5), temperature annual range (BIO 7), mean temperature of wettest quarter (BIO 8), and mean temperature of driest quarter (BIO 9). Average AUC for the subsampled contemporary ENM was 0.90 (±0.01 *SD*) for the training occurrences and 0.89 (±0.01 *SD*) for the test occurrences. The contemporary HS map is reflective of the range‐extent maps generated in the past (Thorp et al., 1983), and in similar HS maps generated by Williams et al. (2014) that estimate the distribution of *B. huntii* with maximum entropy approaches (Figure 2a). Following permutation test of all 10 variables included in the contemporary ENM, it was found that annual mean temperature (BIO 1), temperature seasonality (BIO 2), and average temperature of wettest quarter contributed the 29%, 28%, and 11% to model construction, respectively (Table 2).

**Table 2:** Area under the curve (AUC) environmental niche model (ENM) performance summaries, and bioclimatic variable contribution (%) to the ENM using contemporary bioclimatic data. Italicized values represent permutation importance (%) of a bioclimatic variable. High permutation values suggest high contribution the model in estimating *Bombus huntii* habitat suitability

	Training AUC	Test AUC	AUC STD	BIO 1	BIO 12	BIO 15	BIO 18	BIO 19	BIO 2	BIO 5	BIO 7	BIO 8	BIO 9
Contemporary	0.90	0.89	0.01	29.69	0.55	8.87	9.92	3.74	27.92	0.59	4.44	10.8	3.49
				*37.58*	*1.05*	*10.0*	*2.45*	*4.90*	*21.05*	*1.36*	*10.84*	*6.05*	*4.72*

*Note*

BIO 1 = annual mean temperature, BIO 12 = annual precipitation, BIO 15 = precipitation seasonality, BIO 18 = precipitation of warmest quarter, BIO 19 = precipitation of coldest quarter, BIO 2 = mean diurnal range, BIO 5 = max temperature of warmest month, BIO 7 = temperature annual range, BIO 8 = mean temperature of wettest quarter, BIO 9 = mean temperature of driest quarter.

The contemporary ENM predicts *B. huntii* to be distributed across an elevational gradient throughout the Intermountain West, and primarily at high‐elevation habitats of the Sierra Madre Occidental, Sierra Madre Oriental, and the Trans‐Mexican Volcanic Belt (Figures 1 and 2a). The LGM HS map reveals a dramatic latitudinal shift in the distribution of *B. huntii* HS, where middle latitude mountain ranges relative to the contemporary distribution of the bumble bee are predicted to have been a more suitable environment for *B. huntii* in the cooler Pleistocene (Figure 2b). Based on the LGM map, the Madrean Archipelago is suggested to have possessed the bioclimatic conditions that may have provided *B. huntii* a pathway for dispersal to and from the Sierra Madre Occidental (Figures 1 and 2c).

### 3.2 Hardy–Weinberg equilibirium, linkage disequilibirium, and colony assignment

Of the original 33 sampling sites (*n *=* *380; hereafter refereed to as “populations”), we retained 26 populations (*n *=* *364) for further analyses as they were represented by more than four individuals. In our analyses of the 26 populations, the locus BTERN02 did not amplify in >50% of the specimens genotyped and was removed from any further data processing. MICRO‐CHECKER results indicated that the locus BTMS0059 was suspected to have null alleles in 48% of the populations and was also excluded from further analyses.

B124 showed significant deviation from HWE in the Ada (ADA, *p *=* *0.02) and Baker populations (BAK, *p *<* *0.001), but not in the remaining locus by population combinations (91.3% of the data). BTMS0062 showed significant devation from HWE in the Flagstaff population (FLA, *p *<* *0.001), but not in the remaining locus by population combinations (96.2% of the data). BT28 showed significant deviation from HWE in the Black Hills population (BLH, *p *=* *0.008), but not in the remaining locus by population combinations (95.0% of the data). BT10 showed significant devation from HWE in the Ayahualulco population (AYA, *p* = 0.01), but not in the remaining locus by population combinations (96.2% of the data). Finally, BT30 showed significant devation from HWE in the Ada population (ADA, *p *=* *0.01), but not in the remaining locus by populations combinations (96.0% of the data). Of all the 1,106 locus by population combinations, we detected significant LD only between BTMS0066 and B96 in the Artega‐Galena population (ARG, all *p *<* *0.001). We did not detect any significant LD in any of the remaining 1,105 loci by population combinations (99.9% of the data) after Bonferroni corrections. Given that deviations from HWE or LD are not consistent across any locus by population combination or locus by locus combination, respectively, we elected to retain 11 loci for further sibship and population genetic analyses.

The removal of full‐siblings based on full‐sibship analysis in COLONY v2.0 resulted in the idenfication of 350 colonies of the 364 individuals available for final analysis across the 26 populations (average individuals per population sample = 13.6 ± 1.4 *SE*). Thus, the final sample size used in subsequent analyses reflects the numbers of unrelated individuals (*n *=* *350) available after the COLONY analysis (Table 1). A summary on the number of colonies detected at each of our sites is available in Supporting Information Appendix S3.

### 3.3 Population genetic structure and landscape genetics

**Figure 3:**
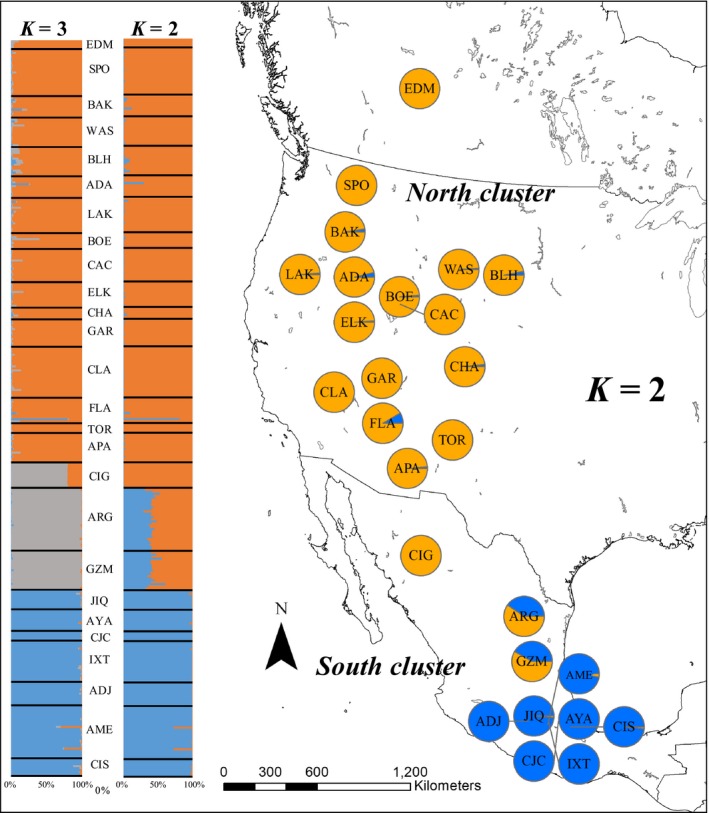
Genetic cluster assignment of 26 *Bombus huntii* populations in western North America inferred with STRUCTURE v 2.3.4 (Pritchard et al., 2000). Pie charts represent fractional assignment probability of each sampling site (i.e., population) to each of the *K *=* *2 genetic clusters. Fractional assignment to the North and South clusters is in orange and blue, respectively. Inset bar graphs represent individual STRUCTURE assignment to each of the *K *=* *2 and *K *=* *3 genetic clusters expressed in decreasing latitude (each bar = 1 individual). Three letter site sampling site codes are defined in Table 1

Based on the admixture ancestry with correlated allele frequencies model in STRUCTURE, the mean log probability of the data was greatest at *K *=* *9 (*LnP*(9)* *= −12,351.01). The Δ*K* statistic (=82.55) was greatest at *K *=* *2, with significantly less explanatory power gained by including additional *K* clusters (Supporting Information Appendix S4). Assuming *K *=* *2 clusters, populations located in the north portion (north of ~28°N) of *B. huntii*'s geographic distribution (Intermountain West + Black Hills [BLH site] + Aspen Park [EDM site] + Sierra Nevada Occidental [CIG site]) are chiefly assigned to the “north cluster” (Figure 3). Populations located in the south portion (south of ~28°N) of *B. huntii*'s geographic range (Trans‐Mexican Volcanic Range) are chiefly assigned to the “south cluster” (Figure 3). The two populations located in the Sierra Nevada Oriental (ARG + GZM) show admixture between the north and south genetic clusters (Figure 3). Assuming *K *=* *3 clusters (Δ*K* = 23.79), fractional genotype assignment associated with the Cidudad Guerrero (CIG) population (>70% fractional assignment) formed a distinct cluster with Artega‐Galena (ARG) and General Zaragoza‐Miquiuana (GZM) (>95% fractional assignment) (Figure 3). Under the *K *=* *3 scenario, the three populations are situated in the Sierra Nevada Occidental (CIG) and Sierra Nevada Oriental (ARG, GZM) (Figures 1 and 2).

**Figure 4:**
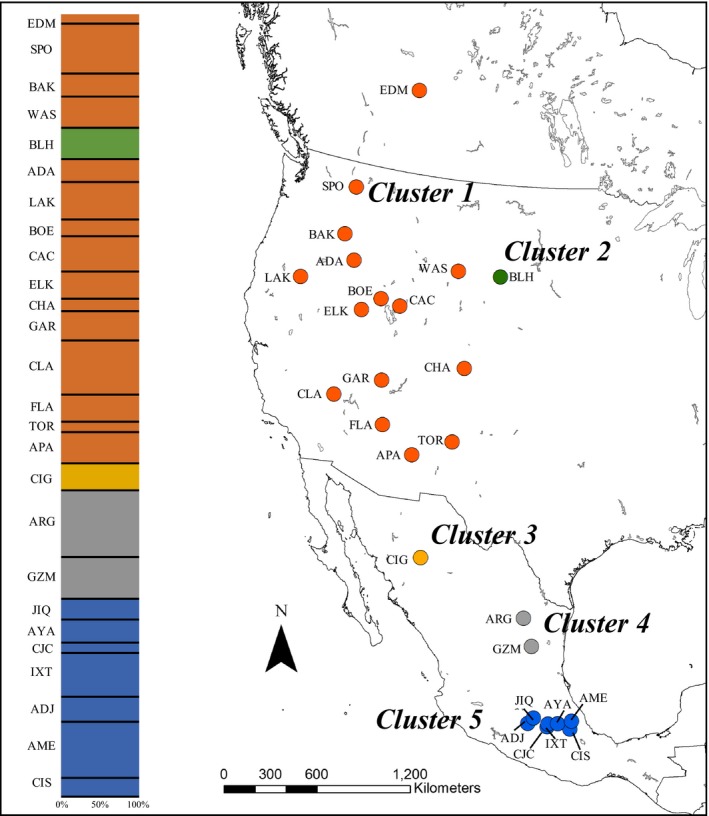
Genetic cluster assignment of 26 *Bombus huntii* populations in western North America inferred with GENELAND v4.05 (Guillot et al., 2012). Cluster 1 = orange, Cluster 2 = green, Cluster 3 = golden yellow, Cluster 4 = gray, and Cluster 5 = blue. Inset bar graphs represent individual GENELAND assignment to each of the *K *=* *5 genetic clusters expressed in decreasing latitude (each bar = 1 individual). Three letter site sampling site codes are defined in Table 1

The inclusion of geographic location information into a population structure analyses with GENELAND estimates *K *=* *5 (Figure 4). The estimate of *K *=* *5 clusters is based on achieving the highest log likelihood value (=−9,225.31, 100 burnin) of all the iteration (1–10) and burnin combinations (100–500) (Supporting Information Appendix S5). Under the *K *=* *5 scenario, the GENELAND model identified populations in the Intermountain West (=14 populations) + Aspen Park ecoregion (EDM) to form cluster 1, the Black Hills population (BLH) to form cluster 2, the Ciudad Guerrero (CIG) population to form cluster 3 (Sierra Nevada Occidental), Artega‐Galena (ARG) and General Zaragoza‐Miquiuana (GZM) populations to form cluster 4 (Sierra Nevada Oriental), and the seven populations found in Trans‐Mexican Volcanic Belt to form cluster 5 (Figure 4).

**Figure 5:**
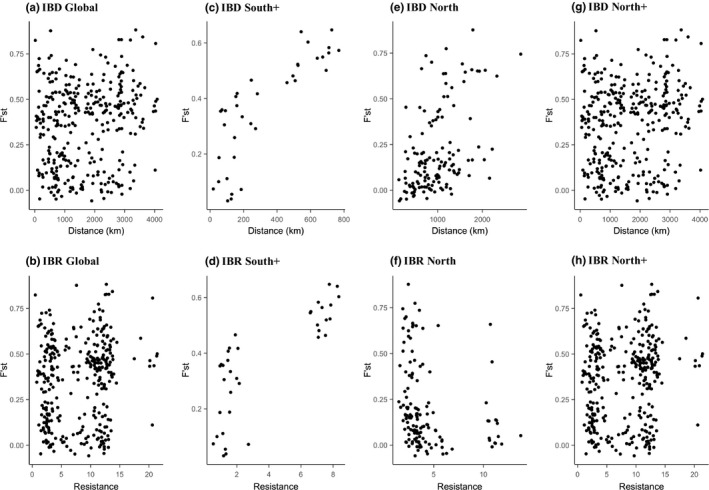
Isolation by geographic distance (IBD) and isolation by environmental resistance (IBR) across 26 *Bombus huntii* populations: (a) IBD Global and (b) IBR Global examines IBD or IBR across all 26 populations regardless of cluster assignment; (c) IBD South+ and (d) IBR South+ examine IBD or IBR across seven southerly distributed populations plus Artega‐Galena (ARG) and General Zaragoza‐Miquiuana (GZM) populations; (e) IBD North and (f) IBR North examine IBD or IBR across 17 northerly distributed populations; and (g) IBD North+ and (h) IBR North+ examine IBD or IBR across 17 northerly distributed populations plus ARG and GZM populations

Examining IBD and resistance patterns with *F*’_ST_ based on STRUCTURE cluster assignment (*K *=* *2) revealed significant differences in population differentiation across samplings sites. Combining all 26 populations (North cluster + South cluster) in a single analysis found IBD to be significant (Mantel test: *r *=* *0.16, *p *=* *0.024) (Figure 5a), but not IBR (Mantel test: *r *=* *0.13, *p *=* *0.111) (Figure 5b). However, while genetic differentiation is significantly correlated with geographic distance (IBD), it should be noted that the strength of the correlation is weak (*r *=* *0.16). We next partitioned the data by STRUCTURE genetic cluster assignment to examine cluster‐specific IBD and IBR models. However, because ARG and GZM were assigned to both north and south clusters (Figure 3), we examined the inclusion of the two populations to either the North or South cluster with IBD and IBR analysis. We found significant IBD (Mantel test: *r *=* *0.82, *p *=* *0.001) (Figure 5c) and IBR (Mantel test: *r *=* *0.80, *p *=* *0.004) (Figure 5d) when including ARG and GZM populations into the South cluster analyses (South+). It should also be noted that the strength of the Mantel correlation is especially high for both IBD and IBR analyses. Excluding ARG and GZM from the South cluster analyses also found significant IBD (Mantel test: *r *=* *0.50, *p *=* *0.02) and IBR (Mantel test: *r *=* *0.50, *p *=* *0.02). However, the Mantel test *r* was much smaller than the South+ models stated above. Excluding ARG and GZM populations from the North cluster analyses found IBD to be significant (Mantel test: *r *=* *0.48, *p *=* *0.013) (Figure 5e), but not IBR (Mantel test: *r *=* *−0.24, *p *=* *0.943) (Figure 5f). Finally, the inclusion of ARG and GZM populations into the North cluster analyses found no significance in IBD (Mantel test: *r *=* *−0.23, *p *=* *0.947) (Figure 5g) and IBR (Mantel test: *r *=* *0.41, *p *=* *0.063) (North+) (Figure 5h). Mantel *r* coefficients and associated *p‐*values for both IBD and IBR models are presented in Table 3. We did not investigate IBD and IBR models using the Geneland genetic cluster assignments as three of the genetic clusters were composed of one or two sampled populations (clusters 2, 3, and 4), and would be an inappropriate sample size for a robust analysis (Figure 4).

**Table 3:** Mantel test summary statistics for four models testing for the effect of geographic distance (IBD) or environmental resistance (IBR) across *Bombus huntii* population and within genetic clusters. The + symbol proceeding the model type represents the two populations that were incorporated into tests of IBD and IBR within a Bayesian inferred genetic cluster. The two additional populations include Artega‐Galena (ARG) and General Zaragoza‐Miquiuana (GZM). Limits presented here represent the 2.5% and 97.5% of the Mantel correlation coefficient *r*. Significance of each model is set at an alpha level of 0.05. Global models include all 26 *B. huntii* populations in the testing for the effect geographic distance or environmental resistance. The IBR model is based on the contemporary HS map of *B. huntii*

Model	Model type	Mantel *r*	*p*	Lower limit (2.5%)	Upper limit (97.5%)
Global	IBD	0.16	0.024	0.10	0.24
	IBR	0.13	0.111	0.06	0.21
South	IBD	0.50	0.021	0.29	0.77
	IBR	0.50	0.018	0.28	0.71
South+	IBD	0.82	0.001	0.78	0.87
	IBR	0.80	0.004	0.74	0.85
North	IBD	0.48	0.013	0.25	0.60
	IBR	−0.24	0.943	−0.37	−0.20
North+	IBD	−0.23	0.947	−0.30	−0.15
	IBR	0.41	0.063	0.32	0.61

### 3.4 Population genetic diversity

**Figure 6:**
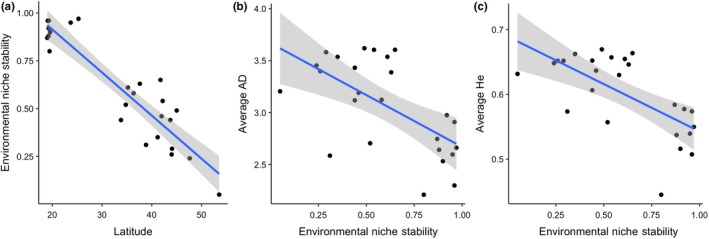
(a) Scatterplot and linear models examining the relationship between latitude and environmental niche stability (ENS) of *Bombus huntii*, (b) the relationship between *B. huntii* effective allelic diversity (*AD*) and ENS, and (c) the relationship between *B. huntii* expected heterozygosity (*H*_e_) and ENS. ENS represents the sum of contemporary (1950–2000) and the last glacial maximum (LGM) habitat suitability (0–1) values predicted by a contemporary ENM (see Figure 3). Stability values closer to 1 represent high HS stability, whereas values closer to 0 represent low HS stability (i.e., unstable)

A preliminary assessment of the relationship between ENS and average *AD* and *H*_e_ found one population, Ciudad Guerrero (CIG), to be a sample outlier relative to the other 25 *B. huntii* populations. The CIG population is associated with low average *AD* (=1.41 ± 0.46 *SE*) and low average *H*_e_ (=0.37 ± 0.11 *SE*) (Table 2). Removing the CIG population from subsequent linear regression analyses increased the ability for ENS to explain the variance in the *AD* and *H*_e_ linear regression models by 147% and 139%, respectively. We found a significant and negative relationship between latitude and ENS across the *B. huntii* populations (*R* ^2^ = 0.84, *F*(1, 23) = 123.5, *p *<* *0.001) (Figure 6a). Because latitude is a significant predictor of ENS, we do not consider latitude in subsequent models examining the effect of ENS on genetic diversity. ENS is a significant and negative predictor of both average *AD* (*R* ^2^ = 0.37, *F*(1, 23) = 13.62, *p *=* *0.001) (Figure 6b) and average *H*_e_ (*R* ^2^ = 0.43, *F*(1, 23) = 17.55, *p *<* *0.001) (Figure 6c).

### 3.5 Environmental niche comparisons

**Figure 7:**
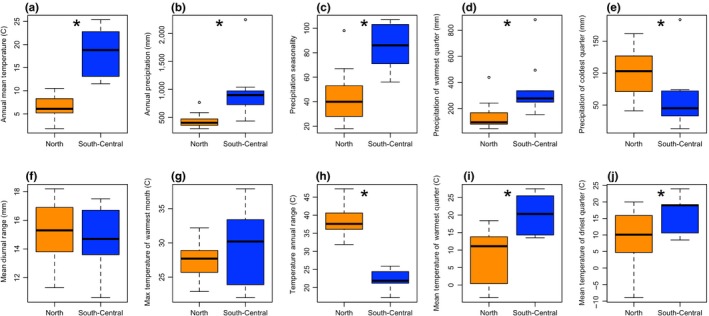
Boxplot visualization of contemporary (1950–2000) environmental niche space occupied by contemporary North and South *Bombus huntii* genetic clusters for 10 bioclimatic variables. Significant differences between the North and South+ populations are tested with a Wilcoxon rank‐sum test and identified with a * symbol at the midline of each graph (alpha = 0.05)

Pairwise comparisons between the bioclimatic variables found that eight of the ten variables used to construct the ENMs exhibited significant differences between the North and South+ genetic clusters of *B. huntii* (Figure 7a–j) (Wilcoxon rank‐sum tests, all *p *<* *0.05). We found no difference between the North and South+ genetic clusters in measurements of contemporary mean diurnal range (Wilcoxon rank‐sum test, *p *=* *0.40) (Figure 7f) and max temperature of warmest month (Wilcoxon rank‐sum test, *p *=* *0.22) (Figure 7g). Average temperature associated with populations in the South+ cluster is significantly more mesic year‐round when compared to temperatures associated with populations in the North cluster (Figure 7h). Furthermore, precipitation seasonality associated with populations in the South+ cluster suggests that there is little seasonal variability, whereas the populations in the North are found in habitats with high seasonal variability (Figure 7c).

## 4 DISCUSSION

Our population genetic study of *B. huntii* found that populations in regions that have been climatically unstable since the LGM (high‐latitude environments, e.g., north of 28°N) exhibit high genetic diversity, whereas regions that have been climatically stable since the LGM (low‐latitude environments, e.g., south of 28°N) exhibit low genetic diversity (Figure 6b–c). Throughout its northerly distribution, *B. huntii* population genetic differentiation is significantly correlated with geographic distance (IBD, Figure 6e), but not bioclimatic resistance (IBR, Figure 6f). Nonsignificant IBR suggests that northern populations of *B. huntii* are not strongly inhibited by environmental heterogeneity, except at long distances between populations (>2,000 km). Populations distributed throughout the north genetic cluster are associated with much colder annual temperatures, a pattern that is certainly correlated with increasing latitude (Figure 3b). Furthermore, an examination of the elevation profile of *B. huntii* ENM in the northern part of its range suggests high HS across a broad elevation gradient (Figure 3b). The broad bioclimatic profile and geographic distribution of *B. huntii* in northern populations likely facilitate range‐wide gene flow (Figures 4a and 6e).

Weak genetic structure and lack of IBD across a large geographic scale are not unique to northern *B. huntii* populations. Several other broadly distributed bumble bee species are found to exhibit weak genetic structure (i.e., low pairwise population *F*’_ST_, and its analogs) at distances greater than >500 km (Lozier et al., 2011). The ability of bumble bees to disperse and forage on a diverse array of pollen sources at great distance is a significant factor in facilitating gene flow across populations (Goulson & Stout, 2001; Jha, 2015; Knight et al., 2005; Moreira, Horgan, Murray, & Kakouli‐Duarte, 2015). Bumble bees are dependent on suitable forage and nesting resources, however, dispersal as a mechanism in facilitating gene flow is limited to the reproductive caste, namely queens and males. While some North American bumble bees exhibit low admixture or population genetic differentiation, other, mostly montane species have been found to exhibit significant IBD and IBR (Hines & Williams, 2012; Jha & Kremen, 2013; Lozier et al., 2013). For example, *B. bifarius* is primarily associated with montane environments throughout its distribution. However, *B. bifarius* also occur at low‐elevation and offshore islands throughout the Pacific Northwest and Central Coast of California (Koch et al., 2012; Williams et al., 2014). These populations have reduced population genetic diversity and exhibit a degree of phenotypic divergence relative to populations distributed across the Colorado Rocky Mountains, the Sierra Nevada, Cascade, Sawtooth, Bighorn, and Uinta Mountains of western North America (Lozier et al., 2013; Lozier et al.*,* 2016). The variability in HS across these montane provinces has been a major barrier to gene flow, revealing significant IBR patterns across *B. bifarius* populations.

Unlike northern *B. huntii* populations, southern populations appear to be limited to high‐elevation habitats throughout the Sierra Madre Oriental and the Trans‐Mexican Volcanic Belt (Figure 2b) and form a distinct genetic cluster (Figure 3). Further examination of population genetic structure with GENELAND suggests three distinct clusters inhabiting Mexico: Sierra Madre Occidental (CIG site), Sierra Madre Oriental (ARG + GZM sites), and the Trans‐Mexican Volcanic belt (=7 sites) (Figure 4). Unlike the STRUCTURE analysis, the GENELAND analysis parameterizes the Bayesian model to include geographic location a priori to model construction. Despite populations being relatively close together (in comparison with pairwise geographic distance observed in northern populations), the south genetic cluster was found to exhibit strong genetic differentiation (*F*’_ST_) across populations (Figure 5c–d). In fact, geographic distance and resistance appear to explain a significant proportion of the variance in *F*’_ST_ estimates in the southern genetic cluster (Table 3). Comparable significance of the IBR model to the IBD model across the southern genetic cluster implies that adjacent environments of contemporary *B. huntii* populations are a major barrier to dispersal, and ultimately gene flow. Examination of the ENMs associated with LGM and contemporary HS suggest that high‐elevation montane environments south of 28°N are more suitable for *B. huntii* than low‐elevation environments (Figure 2a–b). Furthermore, the patterns of genetic structure uncovered in Mexican *B. huntii* populations as they relate to their distribution across the different mountain provinces are like the genetic patterns uncovered by Duennes et al. (2017) in the *B. epphipiatus* species complex. Our study corroborates contemporary investigations into how the complex climate history of the Quaternary, along with the geographic isolation to mountain provinces, has played a significant role in population divergence and cladogensis in Mesoamerican biodiversity.

### 4.1 Quaternary climate variability and population genetic patterns

Pleistocene climate variability has influenced contemporary patterns of biodiversity observed throughout North America (Hewitt, 2000). Specifically, the oscillation between cooling and warming periods is implicated to have driven the dynamics of species and communities distributed throughout montane environments in plants (Callahan et al., 2013) and mammals (Galbreath et al., 2009, 2010), as well as their underlying patterns of genetic diversity (Hewitt, 1996). The correlation between ENS and latitude, along with increased population genetic diversity with decreasing ENS, suggests that northern populations are part of the leading edge of the species range, whereas the more genetically differentiated southern populations are part of the rear edge of the species range (Figure 3) (Hampe & Petit, 2005). The rear edge is defined as the low‐latitude margin of a species’ geographic distribution, whereas the leading edge is defined as the high‐latitude margin of a species’ geographic distribution (Figure 2a). Biogeographic theory predicts that stable rear edge populations occur in regions that would have provided suitable conditions for species to persist during warming and cooling stages of the Quarterary (Hampe & Petit, 2005). Our ENS map predicts that low‐latitude regions, especially the Trans‐Mexican Volcanic Belt and Sierra Madre Occidental, have likely served as climatic refugia for *B. huntii* over the past 22,000 years (Figure 2c).

In addition to relative climate stability, stable rear edge populations are associated with heterogeneous topography that are typically restricted to habitat islands (i.e., sky islands) within a matrix of unsuitable environments (Hampe & Petit, 2005). IBD and IBR models of *B. huntii* populations in southern genetic cluster show that geographic distance and environmental resistance explain a large portion of the variability found in pairwise estimates of genetic differentiation (Figure 5c–d). Finally, reduced within‐population genetic diversity pattern and high genetic differentiation, despite close geographic proximity, are characteristic of stable rear edge populations. Both features of genetic variability (diversity and structure) are readily observed in the patterns of allelic diversity and expected heterozygosity uncovered in our study. We found that high‐latitude populations (leading edge) are associated with high within‐population genetic diversity, and low‐latitude populations (rear edge) are associated with reduced within‐population genetic diversity (Figure 6b–c).

## 5 CONCLUSION

A wealth of phylogeographic studies supports the hypothesis that Quaternary climate variability has shaped range‐wide patterns of population genetic diversity and divergence that are observed in extant populations and species. In our study, we discover clear geographic and environmental differences in the contemporary *B. huntii* populations that are predicated on ENS since the LGM. The major findings of this study include the following: (a) the *B. huntii* ENS map predicts low‐latitude environments to be more climatically stable in comparison with high‐latitude environments over the past 22,000 years; (b) there are at least two genetic clusters (north and south) that a population can be assigned to based on a priori Bayesian analysis, with further evidence for genetic subdivision across Mexican populations found across different mountain ranges; (c) IBD and IBR models are significant across populations in the south genetic cluster, whereas only IBD is significant in the north genetic cluster; (d) genetic diversity increases with latitude and, therefore, decreases within increasing ENS; and (e) there are significant bioclimatic differences between the north and south genetic clusters, with southern populations inhabiting mesic environments with low variation in precipitation across seasons.

The results of this study contribute to our understanding of how historic and contemporary climate may have shaped the patterns of genetic variability observed in insects, an understudied group of animals, relative to vertebrate organisms. Bumble bees are a valuable model to study how climate variability affects patterns of genetic variability as they are highly sensitive to recent climate change. Over the past century, bumble bee populations have responded to a warming climate by emerging earlier in the spring, evolved shorter proboscis, and potentially shifted in latitudinal distribution (Bartomeus et al., 2011; Kerr et al., 2015; Miller‐Struttman et al.*,* 2015). Documenting contemporary patterns of genetic variability has the potential to inform management and conservation policy for both wild and managed bumble bee pollinator populations.

## CONFLICT OF INTEREST

None declared.

## AUTHOR CONTRIBUTIONS

J.B.K. and J.P.S. conceived the idea; J.B.K., J.P.S., R.V., J.M‐R., and P.S., collected the specimens; J.B.K. generated and analyzed the genetic data, and led the writing of the manuscript. J.P.S. and R.V. provided editorial feedback.

## DATA ACCESSIBILITY

Microsatellite genotypes and collection locations are available on FigShare (https://figshare.com/articles/Kochetal_BhuntiiMicroSats_xlsx/6214127) under a CC BY 4 license. Georeferenced records and associated WorldClim variables are available on FigShare (https://figshare.com/articles/1035_georeferenced_locality_records_and_associated_Worldclim_bioclimatic_variables_for_North_American_Bombus_huntii/6214331) under a CC BY 4 license.
