# Supplementary material for: Quaternary climate instability is correlated with patterns of population genetic variability in Bombus huntii
Source: Ecol Evol. 2018 Jul 13;8(16):7849–64. doi: 10.1002/ece3.4294 (PMC6145020; doi:10.1002/ece3.4294)
Supplement: Supplementary file 1 [file ECE3-8-7849-s001.docx]

| **Appendix 1.** Data providers of the georeferenced occurrence records used to construct habitat suitability models and characterize bioclimatic niche of *Bombus huntii*. Data were accessed on 28 June 2015. | |
| --- | --- |
|  | |
|  | **Institution** |
| 1 | iNaturalist.org: iNaturalist research-grade observations doi:10.15468/ab3s5x |
| 2 | USDA-ARS Bee Biology and Systematics Laboratory: Bee Biology and Systematics Laboratory doi:10.15468/anyror |
| 3 | Bombus of Canada doi:10.15468/ip9oon |
| 4 | Museum of Biological Diversity, The Ohio State University: C.A. Triplehorn Insect Collection (OSUC), Ohio State University doi:10.15468/efb17f |
| 5 | Luis Martínez, M. A. 2001. Computarización de la colección de abejas (Hymenoptera: Apoidea) del Museo de Zoología Alfonso L. Herrera, de la Facultad de Ciencias de la UNAM. Universidad Nacional Autónoma de México. Facultad de Ciencias. Bases de datos SNIB2010-CONABIO. Proyecto No. Q035. México, D.F. doi:10.15468/wzfnf2 |
| 6 | University of Colorado Museum of Natural History: UCMC_Entomology doi:10.15468/jsgtns |
| 7 | Biodiversity Institute of Ontario (UBCZ) from University of Guelph. http://dx.doi.org/10.5886/qzxxd2pa (accessed on [date]), doi:10.5886/qzxxd2pa doi:10.5886/qzxxd2pa |
| 8 | Yale University Peabody Museum: Entomology Division, Yale Peabody Museum doi:10.15468/95waq3 |
| 9 | USDA-ARS Bee Biology and Systematics Laboratory: Patterns of widespread decline in North American bumble bees doi:10.15468/kjpwz1 |
| 10 | iNaturalist.org: iNaturalist research-grade observations doi:10.15468/ab3s5x |
| 11 | Illinois Natural History Survey: Illinois Natural History Survey doi:10.15468/eol0pe |
| 12 | University of Kansas Biodiversity Institute: Snow Entomological Museum Collection doi:10.15468/fhntpy |
