# Supplementary material for: Quaternary climate instability is correlated with patterns of population genetic variability in Bombus huntii
Source: Ecol Evol. 2018 Jul 13;8(16):7849–64. doi: 10.1002/ece3.4294 (PMC6145020; doi:10.1002/ece3.4294)
Supplement: Supplementary file 2 [file ECE3-8-7849-s002.docx]

**Appendix 2.** Estimated mistyping rates of each microsatellite locus across species and sites estimated with Colony v2.0 (Jones and Wang 2010).

| **MarkerID** | **B124** | **BTERN01** | **BT28** | **BT10** | **BT30** | **B96** | **BTMS81** | **BTMS0066** | **BTMS0062** | **BL13** | **BTMS0044** |
| --- | --- | --- | --- | --- | --- | --- | --- | --- | --- | --- | --- |
| Ada | 0.00 | 0.13 | 0.00 | 0.11 | 0.06 | 0.00 | 0.01 | 0.05 | 0.22 | 0.01 | 0.00 |
| Almoloya de Juarez | 0.00 | 0.00 | 0.04 | 0.11 | 0.00 | 0.05 | 0.00 | 0.00 | 0.00 | 0.00 | 0.00 |
| Amemaca | 0.00 | 0.00 | 0.00 | 0.00 | 0.04 | 0.00 | 0.00 | 0.00 | 0.04 | 0.00 | 0.00 |
| Apache | 0.00 | 0.10 | 0.02 | 0.01 | 0.17 | 0.04 | 0.10 | 0.00 | 0.00 | 0.03 | 0.00 |
| Artega Galena | 0.00 | 0.00 | 0.00 | 0.00 | 0.00 | 0.00 | 0.00 | 0.00 | 0.05 | 0.00 | 0.02 |
| Ayahualulco | 0.00 | 0.00 | 0.00 | 0.00 | 0.00 | 0.00 | 0.05 | 0.00 | 0.07 | 0.03 | 0.00 |
| Baker | 0.00 | 0.00 | 0.00 | 0.00 | 0.00 | 0.00 | 0.00 | 0.00 | 0.00 | 0.00 | 0.00 |
| Black Hills | 0.08 | 0.06 | 0.03 | 0.00 | 0.00 | 0.00 | 0.04 | 0.07 | 0.21 | 0.13 | 0.03 |
| Box Elder | 0.00 | 0.00 | 0.03 | 0.00 | 0.00 | 0.00 | 0.00 | 0.00 | 0.00 | 0.00 | 0.02 |
| Cache | 0.00 | 0.05 | 0.00 | 0.00 | 0.00 | 0.02 | 0.04 | 0.04 | 0.00 | 0.01 | 0.06 |
| Chaffee | 0.00 | 0.00 | 0.00 | 0.01 | 0.10 | 0.00 | 0.00 | 0.00 | 0.00 | 0.11 | 0.00 |
| Ciudad Guerrero | 0.00 | 0.00 | 0.00 | 0.00 | 0.00 | 0.00 | 0.00 | 0.00 | 0.03 | 0.00 | 0.00 |
| Ciudad Serdan | 0.04 | 0.00 | 0.00 | 0.00 | 0.00 | 0.04 | 0.00 | 0.05 | 0.32 | 0.00 | 0.00 |
| Clark | 0.08 | 0.10 | 0.05 | 0.11 | 0.05 | 0.00 | 0.04 | 0.06 | 0.00 | 0.00 | 0.00 |
| Colon | 0.00 | 0.00 | 0.00 | 0.00 | 0.00 | 0.03 | 0.05 | 0.00 | 0.00 | 0.00 | 0.00 |
| Cont la de Juan | 0.07 | 0.00 | 0.00 | 0.06 | 0.00 | 0.15 | 0.00 | 0.00 | 0.00 | 0.11 | 0.06 |
| Edmonton | 0.00 | 0.00 | 0.00 | 0.00 | 0.00 | 0.00 | 0.00 | 0.00 | 0.00 | 0.00 | 0.00 |
| Elko | 0.00 | 0.00 | 0.07 | 0.15 | 0.05 | 0.00 | 0.00 | 0.03 | 0.27 | 0.08 | 0.00 |
| Flagstaff | 0.04 | 0.00 | 0.09 | 0.20 | 0.00 | 0.00 | 0.00 | 0.04 | 0.18 | 0.03 | 0.00 |
| Garfield | 0.00 | 0.15 | 0.00 | 0.01 | 0.00 | 0.13 | 0.02 | 0.00 | 0.00 | 0.00 | 0.03 |
| General Zargosa | 0.02 | 0.00 | 0.00 | 0.00 | 0.02 | 0.00 | 0.04 | 0.03 | 0.00 | 0.00 | 0.02 |
| Ixtapulca | 0.01 | 0.04 | 0.03 | 0.00 | 0.01 | 0.00 | 0.05 | 0.02 | 0.00 | 0.00 | 0.00 |
| Jiquipilco | 0.00 | 0.00 | 0.00 | 0.00 | 0.01 | 0.00 | 0.00 | 0.08 | 0.00 | 0.00 | 0.00 |
| Lake | 0.18 | 0.09 | 0.00 | 0.00 | 0.07 | 0.02 | 0.00 | 0.00 | 0.12 | 0.03 | 0.02 |
| Larimer | 0.00 | 0.00 | 0.00 | 0.00 | 0.00 | 0.00 | 0.00 | 0.00 | 0.00 | 0.00 | 0.00 |
| Spokane | 0.03 | 0.00 | 0.00 | 0.13 | 0.08 | 0.02 | 0.06 | 0.00 | 0.00 | 0.06 | 0.06 |
| Torrance | 0.00 | 0.00 | 0.00 | 0.00 | 0.00 | 0.00 | 0.00 | 0.33 | 0.06 | 0.00 | 0.15 |
| Washakie | 0.00 | 0.06 | 0.00 | 0.05 | 0.04 | 0.00 | 0.00 | 0.00 | 0.03 | 0.00 | 0.00 |
