# Supplementary material for: Quaternary climate instability is correlated with patterns of population genetic variability in Bombus huntii
Source: Ecol Evol. 2018 Jul 13;8(16):7849–64. doi: 10.1002/ece3.4294 (PMC6145020; doi:10.1002/ece3.4294)
Supplement: Supplementary file 3 [file ECE3-8-7849-s003.docx]

**Appendix 3.** Sibship reconstruction across species and sites estimated with Colony v2.0 (Jones and Wang 2010).

| **Population** | **Colony Index** | **Inclusion Probability** | **Exclusion Probability** | **Member Identification** |
| --- | --- | --- | --- | --- |
| Ada | 1 | 1 | 1 | Ada1 |
| Ada | 2 | 1 | 0.9971 | Ada2 |
| Ada | 3 | 1 | 1 | Ada3 |
| Ada | 4 | 1 | 1 | Ada4 |
| Ada | 5 | 1 | 0.9833 | Ada5 |
| Ada | 6 | 1 | 1 | Ada6 |
| Ada | 7 | 1 | 1 | Ada7 |
| Ada | 8 | 1 | 0.9971 | Ada8 |
| Ada | 9 | 1 | 1 | Ada9 |
| Ada | 10 | 1 | 0.9833 | Ada10 |
| Almoloya de Juarez | 1 | 1 | 0.9627 | AlmoloyadeJuarez11 |
| Almoloya de Juarez | 2 | 1 | 0.9426 | AlmoloyadeJuarez12 |
| Almoloya de Juarez | 3 | 1 | 0.9602 | AlmoloyadeJuarez13 |
| Almoloya de Juarez | 4 | 1 | 0.9961 | AlmoloyadeJuarez14 |
| Almoloya de Juarez | 5 | 1 | 0.9389 | AlmoloyadeJuarez15 |
| Almoloya de Juarez | 6 | 1 | 0.9997 | AlmoloyadeJuarez16 |
| Almoloya de Juarez | 7 | 1 | 0.9097 | AlmoloyadeJuarez17 |
| Almoloya de Juarez | 8 | 0.7577 | 0.7577 | AlmoloyadeJuarez18, AlmoloyadeJuarez19, AlmoloyadeJuarez21 |
| Almoloya de Juarez | 9 | 1 | 0.969 | AlmoloyadeJuarez20 |
| Amecameca | 1 | 1 | 0.975 | Amecameca22 |
| Amecameca | 2 | 1 | 0.9943 | Amecameca23 |
| Amecameca | 3 | 1 | 0.6049 | Amecameca24 |
| Amecameca | 4 | 1 | 0.9972 | Amecameca25 |
| Amecameca | 5 | 1 | 1 | Amecameca26 |
| Amecameca | 6 | 0.7491 | 0.7491 | Amecameca27, Amecameca28, Amecameca33 |
| Amecameca | 7 | 1 | 0.9155 | Amecameca29 |
| Amecameca | 8 | 1 | 0.9951 | Amecameca30 |
| Amecameca | 9 | 1 | 0.9592 | Amecameca31 |
| Amecameca | 10 | 1 | 0.9931 | Amecameca32 |
| Amecameca | 11 | 1 | 0.8927 | Amecameca34 |
| Amecameca | 12 | 1 | 0.2734 | Amecameca35 |
| Amecameca | 13 | 0.9964 | 0.9957 | Amecameca36, Amecameca38, Amecameca39 |
| Amecameca | 14 | 1 | 0.976 | Amecameca37 |
| Amecameca | 15 | 1 | 0.9629 | Amecameca40 |
| Amecameca | 16 | 0.9978 | 0.9978 | Amecameca41, Amecameca43, Amecameca44 |
| Amecameca | 17 | 1 | 0.2602 | Amecameca42 |
| Amecameca | 18 | 1 | 0.5796 | Amecameca45 |
| Amecameca | 19 | 1 | 0.8992 | Amecameca46 |
| Amecameca | 20 | 1 | 0.8822 | Amecameca47 |
| Amecameca | 21 | 1 | 0.9155 | Amecameca48 |
| Amecameca | 22 | 1 | 0.9969 | Amecameca49 |
| Amecameca | 23 | 1 | 0.9989 | Amecameca50 |
| Amecameca | 24 | 1 | 0.9972 | Amecameca51 |
| Apache | 1 | 1 | 0.9944 | Apache52 |
| Apache | 2 | 1 | 1 | Apache53 |
| Apache | 3 | 0.8834 | 0.8822 | Apache54, Apache63 |
| Apache | 4 | 1 | 0.4607 | Apache55 |
| Apache | 5 | 1 | 1 | Apache56 |
| Apache | 6 | 1 | 0.9944 | Apache57 |
| Apache | 7 | 1 | 0.9987 | Apache58 |
| Apache | 8 | 1 | 1 | Apache59 |
| Apache | 9 | 1 | 1 | Apache60 |
| Apache | 10 | 1 | 0.9987 | Apache61 |
| Apache | 11 | 1 | 0.4697 | Apache62 |
| Apache | 12 | 1 | 0.9525 | Apache64 |
| Apache | 13 | 1 | 0.8484 | Apache65 |
| Artega-Galena | 1 | 1 | 0.9711 | ArtegaGalena66 |
| Artega-Galena | 2 | 1 | 0.9141 | ArtegaGalena67 |
| Artega-Galena | 3 | 1 | 0.6807 | ArtegaGalena68 |
| Artega-Galena | 4 | 1 | 0.4754 | ArtegaGalena69 |
| Artega-Galena | 5 | 1 | 1 | ArtegaGalena70 |
| Artega-Galena | 6 | 1 | 1 | ArtegaGalena71 |
| Artega-Galena | 7 | 1 | 0.7034 | ArtegaGalena72 |
| Artega-Galena | 8 | 1 | 0.6744 | ArtegaGalena73 |
| Artega-Galena | 9 | 0.9949 | 0.5603 | ArtegaGalena74, ArtegaGalena75 |
| Artega-Galena | 10 | 1 | 0.811 | ArtegaGalena76 |
| Artega-Galena | 11 | 1 | 0.2126 | ArtegaGalena77 |
| Artega-Galena | 12 | 1 | 0.9988 | ArtegaGalena78 |
| Artega-Galena | 13 | 1 | 0.9268 | ArtegaGalena79 |
| Artega-Galena | 14 | 1 | 0.6817 | ArtegaGalena80 |
| Artega-Galena | 15 | 1 | 0.2542 | ArtegaGalena81 |
| Artega-Galena | 16 | 1 | 0.9724 | ArtegaGalena82 |
| Artega-Galena | 17 | 1 | 0.6946 | ArtegaGalena83 |
| Artega-Galena | 18 | 1 | 1 | ArtegaGalena84 |
| Artega-Galena | 19 | 1 | 0.958 | ArtegaGalena85 |
| Artega-Galena | 20 | 1 | 0.8418 | ArtegaGalena86 |
| Artega-Galena | 21 | 1 | 0.8505 | ArtegaGalena87 |
| Artega-Galena | 22 | 1 | 0.3264 | ArtegaGalena88 |
| Artega-Galena | 23 | 1 | 0.4768 | ArtegaGalena89 |
| Artega-Galena | 24 | 1 | 0.6875 | ArtegaGalena90 |
| Artega-Galena | 25 | 1 | 0.7645 | ArtegaGalena91 |
| Artega-Galena | 26 | 1 | 0.8466 | ArtegaGalena92 |
| Artega-Galena | 27 | 1 | 0.7337 | ArtegaGalena93 |
| Artega-Galena | 28 | 1 | 0.9675 | ArtegaGalena94 |
| Artega-Galena | 29 | 1 | 1 | ArtegaGalena95 |
| Artega-Galena | 30 | 1 | 0.9397 | ArtegaGalena96 |
| Artega-Galena | 31 | 1 | 0.9869 | ArtegaGalena97 |
| Ayahualulco | 1 | 0.8144 | 0.3218 | Ayahualulco98, Ayahualulco102 |
| Ayahualulco | 2 | 0.788 | 0.7879 | Ayahualulco99, Ayahualulco105, Ayahualulco106, Ayahualulco107 |
| Ayahualulco | 3 | 1 | 0.9999 | Ayahualulco100 |
| Ayahualulco | 4 | 1 | 0.2428 | Ayahualulco101 |
| Ayahualulco | 5 | 1 | 0.9981 | Ayahualulco103 |
| Ayahualulco | 6 | 1 | 1 | Ayahualulco104 |
| Baker | 1 | 1 | 1 | Baker108 |
| Baker | 2 | 1 | 1 | Baker109 |
| Baker | 3 | 1 | 1 | Baker110 |
| Baker | 4 | 1 | 1 | Baker111 |
| Baker | 5 | 1 | 1 | Baker112 |
| Baker | 6 | 1 | 1 | Baker113 |
| Baker | 7 | 1 | 1 | Baker114 |
| Baker | 8 | 1 | 1 | Baker115 |
| Baker | 9 | 1 | 1 | Baker116 |
| Baker | 10 | 1 | 1 | Baker117 |
| Black Hills | 1 | 1 | 1 | BlackHills118 |
| Black Hills | 2 | 1 | 0.9969 | BlackHills119 |
| Black Hills | 3 | 1 | 0.9861 | BlackHills120 |
| Black Hills | 4 | 1 | 1 | BlackHills121 |
| Black Hills | 5 | 1 | 1 | BlackHills122 |
| Black Hills | 6 | 1 | 0.3923 | BlackHills123 |
| Black Hills | 7 | 1 | 1 | BlackHills124 |
| Black Hills | 8 | 1 | 0.4693 | BlackHills125 |
| Black Hills | 9 | 1 | 0.9861 | BlackHills126 |
| Black Hills | 10 | 1 | 0.9994 | BlackHills127 |
| Black Hills | 11 | 1 | 0.9994 | BlackHills128 |
| Black Hills | 12 | 1 | 0.9422 | BlackHills129 |
| Black Hills | 13 | 1 | 0.9422 | BlackHills130 |
| Black Hills | 14 | 1 | 0.8201 | BlackHills131 |
| Box Elder | 1 | 1 | 1 | BoxElder132 |
| Box Elder | 2 | 1 | 1 | BoxElder133 |
| Box Elder | 3 | 0.9857 | 0.9857 | BoxElder134, BoxElder135 |
| Box Elder | 4 | 1 | 0.9999 | BoxElder136 |
| Box Elder | 5 | 0.9884 | 0.9884 | BoxElder137, BoxElder138 |
| Box Elder | 6 | 1 | 0.9972 | BoxElder139 |
| Box Elder | 7 | 1 | 0.9972 | BoxElder140 |
| Cache | 1 | 1 | 1 | Cache141 |
| Cache | 2 | 1 | 1 | Cache142 |
| Cache | 3 | 1 | 0.9982 | Cache143 |
| Cache | 4 | 1 | 0.9998 | Cache144 |
| Cache | 5 | 1 | 1 | Cache145 |
| Cache | 6 | 1 | 0.9757 | Cache146 |
| Cache | 7 | 0.9935 | 0.9935 | Cache147, Cache154 |
| Cache | 8 | 0.8749 | 0.8749 | Cache148, Cache149 |
| Cache | 9 | 1 | 0.9898 | Cache150 |
| Cache | 10 | 1 | 1 | Cache151 |
| Cache | 11 | 1 | 0.9765 | Cache152 |
| Cache | 12 | 1 | 0.99 | Cache153 |
| Cache | 13 | 1 | 1 | Cache155 |
| Cache | 14 | 1 | 1 | Cache156 |
| Cache | 15 | 1 | 1 | Cache157 |
| Chaffee | 1 | 0.6398 | 0.6398 | Chaffee158, Chaffee160, Chaffee161 |
| Chaffee | 2 | 1 | 0.9993 | Chaffee159 |
| Chaffee | 3 | 1 | 0.9993 | Chaffee162 |
| Ciudad Guerrero | 1 | 1 | 1 | CiudadGuerrero163 |
| Ciudad Guerrero | 2 | 1 | 0.0572 | CiudadGuerrero164 |
| Ciudad Guerrero | 3 | 0.2542 | 0.0509 | CiudadGuerrero165, CiudadGuerrero168 |
| Ciudad Guerrero | 4 | 1 | 0.1179 | CiudadGuerrero166 |
| Ciudad Guerrero | 5 | 1 | 0.0732 | CiudadGuerrero167 |
| Ciudad Guerrero | 6 | 1 | 0.4616 | CiudadGuerrero169 |
| Ciudad Guerrero | 7 | 1 | 0.5443 | CiudadGuerrero170 |
| Ciudad Guerrero | 8 | 1 | 0.2946 | CiudadGuerrero171 |
| Ciudad Guerrero | 9 | 1 | 0.259 | CiudadGuerrero172 |
| Ciudad Guerrero | 10 | 1 | 0.1849 | CiudadGuerrero173 |
| Ciudad Guerrero | 11 | 1 | 0.3833 | CiudadGuerrero174 |
| Ciudad Serdan | 1 | 1 | 0.9989 | CiudadSerdan177 |
| Ciudad Serdan | 2 | 1 | 1 | CiudadSerdan178 |
| Ciudad Serdan | 3 | 0.9964 | 0.9964 | CiudadSerdan179, CiudadSerdan180 |
| Ciudad Serdan | 4 | 1 | 1 | CiudadSerdan181 |
| Ciudad Serdan | 5 | 1 | 1 | CiudadSerdan182 |
| Ciudad Serdan | 6 | 1 | 0.9784 | CiudadSerdan183 |
| Ciudad Serdan | 7 | 1 | 0.9784 | CiudadSerdan184 |
| Ciudad Serdan | 8 | 1 | 0.9989 | CiudadSerdan185 |
| Clark | 1 | 1 | 0.5863 | Clark186 |
| Clark | 2 | 1 | 0.9552 | Clark187 |
| Clark | 3 | 1 | 0.984 | Clark188 |
| Clark | 4 | 1 | 0.9962 | Clark189 |
| Clark | 5 | 1 | 1 | Clark190 |
| Clark | 6 | 1 | 1 | Clark191 |
| Clark | 7 | 1 | 0.9857 | Clark192 |
| Clark | 8 | 1 | 0.9532 | Clark193 |
| Clark | 9 | 1 | 0.9503 | Clark194 |
| Clark | 10 | 1 | 0.9853 | Clark195 |
| Clark | 11 | 1 | 1 | Clark196 |
| Clark | 12 | 1 | 0.944 | Clark197 |
| Clark | 13 | 1 | 0.8948 | Clark198 |
| Clark | 14 | 1 | 0.9757 | Clark199 |
| Clark | 15 | 1 | 0.9909 | Clark200 |
| Clark | 16 | 1 | 0.9534 | Clark201 |
| Clark | 17 | 1 | 0.9971 | Clark202 |
| Clark | 18 | 1 | 1 | Clark203 |
| Clark | 19 | 1 | 1 | Clark204 |
| Clark | 20 | 1 | 0.8839 | Clark205 |
| Clark | 21 | 1 | 0.9858 | Clark206 |
| Clark | 22 | 1 | 0.9989 | Clark207 |
| Clark | 23 | 0.999 | 0.8374 | Clark208, Clark209 |
| Clark | 24 | 1 | 0.9953 | Clark210 |
| Clark | 25 | 1 | 0.9853 | Clark211 |
| Contla de Juann Cuamatzi | 1 | 1 | 0.9985 | Cont215 |
| Contla de Juann Cuamatzi | 2 | 0.1082 | 0.1082 | Cont216, Cont217 |
| Contla de Juann Cuamatzi | 3 | 1 | 0.9999 | Cont218 |
| Edmonton | 1 | 1 | 1 | Edmonton219 |
| Edmonton | 2 | 1 | 0.9993 | Edmonton220 |
| Edmonton | 3 | 1 | 0.9993 | Edmonton221 |
| Edmonton | 4 | 1 | 1 | Edmonton222 |
| Elko | 1 | 1 | 1 | Elko223 |
| Elko | 2 | 1 | 1 | Elko224 |
| Elko | 3 | 1 | 1 | Elko225 |
| Elko | 4 | 1 | 1 | Elko226 |
| Elko | 5 | 1 | 1 | Elko227 |
| Elko | 6 | 1 | 1 | Elko228 |
| Elko | 7 | 1 | 1 | Elko229 |
| Elko | 8 | 1 | 1 | Elko230 |
| Elko | 9 | 1 | 0.9644 | Elko231 |
| Elko | 10 | 1 | 0.9644 | Elko232 |
| Elko | 11 | 1 | 1 | Elko233 |
| Elko | 12 | 1 | 1 | Elko234 |
| Flagstaff | 1 | 1 | 1 | Flagstaff235 |
| Flagstaff | 2 | 1 | 1 | Flagstaff236 |
| Flagstaff | 3 | 1 | 1 | Flagstaff237 |
| Flagstaff | 4 | 1 | 0.9965 | Flagstaff238 |
| Flagstaff | 5 | 1 | 0.9683 | Flagstaff239 |
| Flagstaff | 6 | 1 | 0.9683 | Flagstaff240 |
| Flagstaff | 7 | 1 | 0.9965 | Flagstaff241 |
| Flagstaff | 8 | 1 | 1 | Flagstaff242 |
| Flagstaff | 9 | 1 | 1 | Flagstaff243 |
| Flagstaff | 10 | 1 | 0.9978 | Flagstaff244 |
| Flagstaff | 11 | 1 | 0.6955 | Flagstaff245 |
| Flagstaff | 12 | 1 | 0.697 | Flagstaff246 |
| Garfield | 1 | 1 | 0.8956 | Garfield247 |
| Garfield | 2 | 0.8743 | 0.8743 | Garfield248, Garfield252 |
| Garfield | 3 | 1 | 0.9991 | Garfield249 |
| Garfield | 4 | 1 | 1 | Garfield250 |
| Garfield | 5 | 0.9944 | 0.9944 | Garfield251, Garfield260 |
| Garfield | 6 | 1 | 1 | Garfield253 |
| Garfield | 7 | 1 | 1 | Garfield254 |
| Garfield | 8 | 1 | 0.9999 | Garfield255 |
| Garfield | 9 | 1 | 1 | Garfield256 |
| Garfield | 10 | 1 | 0.9086 | Garfield257 |
| Garfield | 11 | 1 | 0.9846 | Garfield258 |
| Garfield | 12 | 1 | 1 | Garfield259 |
| General Zaragoza-Miquiuana | 1 | 1 | 0.9807 | GZM61 |
| General Zaragoza-Miquiuana | 2 | 1 | 0.9638 | GZM262 |
| General Zaragoza-Miquiuana | 3 | 1 | 1 | GZM263 |
| General Zaragoza-Miquiuana | 4 | 1 | 0.9143 | GZM264 |
| General Zaragoza-Miquiuana | 5 | 1 | 0.27 | GZM265 |
| General Zaragoza-Miquiuana | 6 | 1 | 0.2699 | GZM266 |
| General Zaragoza-Miquiuana | 7 | 1 | 0.9061 | GZM267 |
| General Zaragoza-Miquiuana | 8 | 1 | 0.7346 | GZM268 |
| General Zaragoza-Miquiuana | 9 | 0.9933 | 0.9874 | GZM269, GZM270 |
| General Zaragoza-Miquiuana | 10 | 1 | 0.9332 | GZM271 |
| General Zaragoza-Miquiuana | 11 | 1 | 0.8414 | GZM272 |
| General Zaragoza-Miquiuana | 12 | 1 | 0.7845 | GZM273 |
| General Zaragoza-Miquiuana | 13 | 1 | 0.9883 | GZM274 |
| General Zaragoza-Miquiuana | 14 | 1 | 0.634 | GZM275 |
| General Zaragoza-Miquiuana | 15 | 1 | 0.9577 | GZM276 |
| General Zaragoza-Miquiuana | 16 | 1 | 1 | GZM277 |
| General Zaragoza-Miquiuana | 17 | 1 | 0.8984 | GZM278 |
| General Zaragoza-Miquiuana | 18 | 1 | 1 | GZM279 |
| General Zaragoza-Miquiuana | 19 | 1 | 0.9459 | GZM280 |
| Ixtapaluca | 1 | 1 | 1 | Ixtapaluca281 |
| Ixtapaluca | 2 | 1 | 0.9283 | Ixtapaluca282 |
| Ixtapaluca | 3 | 1 | 0.8234 | Ixtapaluca283 |
| Ixtapaluca | 4 | 1 | 0.9726 | Ixtapaluca284 |
| Ixtapaluca | 5 | 1 | 0.6785 | Ixtapaluca285 |
| Ixtapaluca | 6 | 1 | 0.9921 | Ixtapaluca286 |
| Ixtapaluca | 7 | 1 | 1 | Ixtapaluca287 |
| Ixtapaluca | 8 | 1 | 0.9877 | Ixtapaluca288 |
| Ixtapaluca | 9 | 1 | 0.7653 | Ixtapaluca289 |
| Ixtapaluca | 10 | 1 | 0.858 | Ixtapaluca290 |
| Ixtapaluca | 11 | 1 | 0.9705 | Ixtapaluca291 |
| Ixtapaluca | 12 | 1 | 0.9832 | Ixtapaluca292 |
| Ixtapaluca | 13 | 1 | 0.7996 | Ixtapaluca293 |
| Ixtapaluca | 14 | 1 | 0.9106 | Ixtapaluca294 |
| Ixtapaluca | 15 | 0.9384 | 0.9384 | Ixtapaluca295, Ixtapaluca296, Ixtapaluca298, Ixtapaluca299 |
| Ixtapaluca | 16 | 1 | 0.9054 | Ixtapaluca297 |
| Ixtapaluca | 17 | 1 | 1 | Ixtapaluca300 |
| Jiquipilco | 1 | 1 | 0.7877 | Jiquipilco301 |
| Jiquipilco | 2 | 1 | 0.9997 | Jiquipilco302 |
| Jiquipilco | 3 | 1 | 0.9999 | Jiquipilco303 |
| Jiquipilco | 4 | 0.9963 | 0.9963 | Jiquipilco304, Jiquipilco305 |
| Jiquipilco | 5 | 1 | 0.7942 | Jiquipilco306 |
| Jiquipilco | 6 | 1 | 0.9999 | Jiquipilco307 |
| Jiquipilco | 7 | 0.9023 | 0.9023 | Jiquipilco308, Jiquipilco310 |
| Jiquipilco | 8 | 1 | 0.9911 | Jiquipilco309 |
| Lake | 1 | 1 | 1 | Lake311 |
| Lake | 2 | 1 | 1 | Lake312 |
| Lake | 3 | 1 | 0.9418 | Lake313 |
| Lake | 4 | 1 | 0.9987 | Lake314 |
| Lake | 5 | 1 | 0.9998 | Lake315 |
| Lake | 6 | 1 | 0.9821 | Lake316 |
| Lake | 7 | 1 | 1 | Lake317 |
| Lake | 8 | 1 | 1 | Lake318 |
| Lake | 9 | 1 | 0.997 | Lake319 |
| Lake | 10 | 1 | 0.9771 | Lake320 |
| Lake | 11 | 1 | 1 | Lake321 |
| Lake | 12 | 1 | 0.9822 | Lake322 |
| Lake | 13 | 1 | 0.997 | Lake323 |
| Lake | 14 | 1 | 1 | Lake324 |
| Lake | 15 | 1 | 1 | Lake325 |
| Lake | 16 | 1 | 0.9771 | Lake326 |
| Lake | 17 | 1 | 0.9431 | Lake327 |
| Spokane | 1 | 1 | 0.9858 | Spokane335 |
| Spokane | 2 | 1 | 0.9864 | Spokane336 |
| Spokane | 3 | 1 | 1 | Spokane337 |
| Spokane | 4 | 1 | 0.8939 | Spokane338 |
| Spokane | 5 | 1 | 0.992 | Spokane339 |
| Spokane | 6 | 1 | 1 | Spokane340 |
| Spokane | 7 | 1 | 1 | Spokane341 |
| Spokane | 8 | 1 | 0.9991 | Spokane342 |
| Spokane | 9 | 1 | 0.9059 | Spokane343 |
| Spokane | 10 | 1 | 1 | Spokane344 |
| Spokane | 11 | 1 | 0.9327 | Spokane345 |
| Spokane | 12 | 1 | 0.9859 | Spokane346 |
| Spokane | 13 | 1 | 0.973 | Spokane347 |
| Spokane | 14 | 1 | 0.8461 | Spokane348 |
| Spokane | 15 | 1 | 0.8328 | Spokane349 |
| Spokane | 16 | 1 | 0.985 | Spokane350 |
| Spokane | 17 | 1 | 0.991 | Spokane351 |
| Spokane | 18 | 1 | 0.9923 | Spokane352 |
| Spokane | 19 | 1 | 0.9991 | Spokane353 |
| Spokane | 20 | 1 | 0.9902 | Spokane354 |
| Spokane | 21 | 1 | 0.9961 | Spokane355 |
| Spokane | 22 | 1 | 0.9912 | Spokane356 |
| Spokane | 23 | 1 | 1 | Spokane357 |
| Torrance | 1 | 1 | 0.9999 | Torrance358 |
| Torrance | 2 | 1 | 0.5496 | Torrance359 |
| Torrance | 3 | 1 | 0.5497 | Torrance360 |
| Torrance | 4 | 1 | 1 | Torrance361 |
| Washakie | 1 | 1 | 1 | Washakie362 |
| Washakie | 2 | 1 | 1 | Washakie363 |
| Washakie | 3 | 1 | 1 | Washakie364 |
| Washakie | 4 | 1 | 0.9992 | Washakie365 |
| Washakie | 5 | 1 | 0.9984 | Washakie366 |
| Washakie | 6 | 0.9997 | 0.9997 | Washakie367, Washakie375 |
| Washakie | 7 | 1 | 1 | Washakie368 |
| Washakie | 8 | 1 | 0.9893 | Washakie369 |
| Washakie | 9 | 1 | 0.9984 | Washakie370 |
| Washakie | 10 | 1 | 0.9479 | Washakie371 |
| Washakie | 11 | 1 | 0.9486 | Washakie372 |
| Washakie | 12 | 1 | 0.9893 | Washakie373 |
| Washakie | 13 | 1 | 1 | Washakie374 |
| Washakie | 14 | 1 | 1 | Washakie376 |
