# Supplementary material for: Quaternary climate instability is correlated with patterns of population genetic variability in Bombus huntii
Source: Ecol Evol. 2018 Jul 13;8(16):7849–64. doi: 10.1002/ece3.4294 (PMC6145020; doi:10.1002/ece3.4294)
Supplement: Supplementary file 4 [file ECE3-8-7849-s004.docx]

**Appendix 4.** Four model fit probabilities implemented with the Evanno Method associated with different values of *K* (i.e., clusters) based on 11 microsatellites implemented in STRUCTURE Harvester. Bold italic text represents the indices that suggests the value of *K* that best predicts the microsatellite genotypes assigned in the STRUCTURE analysis. High values of Delta K suggest better fit of the genotype data to the number of proposed *K*.

| **K** | **Reps** | **Mean LnP(K)** | **Stdev LnP(K)** | **Ln'(K)** | **\|Ln''(K)\|** | **Delta K** |
| --- | --- | --- | --- | --- | --- | --- |
| 1 | 10 | -14953.2 | 0.5944 | NA | NA | NA |
| ***2*** | ***10*** | ***-13730.47*** | ***6.4557*** | ***1222.73*** | ***532.82*** | ***82.535206*** |
| 3 | 10 | -13040.56 | 15.2042 | 689.91 | 361.68 | 23.788194 |
| 4 | 10 | -12712.33 | 21.8627 | 328.23 | 66.19 | 3.027537 |
| 5 | 10 | -12450.29 | 100.5978 | 262.04 | 186.21 | 1.851035 |
| 6 | 10 | -12374.46 | 227.717 | 75.83 | 52.38 | 0.230022 |
| 7 | 10 | -12351.01 | 145.8636 | 23.45 | 10.89 | 0.074659 |
| 8 | 10 | -12338.45 | 65.8227 | 12.56 | 17.25 | 0.262068 |
| 9 | 10 | -12308.64 | 57.4257 | 29.81 | 54.1 | 0.942086 |
| 10 | 10 | -12332.93 | 36.6872 | -24.29 | NA | NA |
