# Supplementary material for: Quaternary climate instability is correlated with patterns of population genetic variability in Bombus huntii
Source: Ecol Evol. 2018 Jul 13;8(16):7849–64. doi: 10.1002/ece3.4294 (PMC6145020; doi:10.1002/ece3.4294)
Supplement: Supplementary file 5 [file ECE3-8-7849-s005.docx]

**Appendix 5.** Log probability for different *K* clusters (*K* = 1 - 10) based on different iteration burin values using the GENELAND v4.05 (Guillot et al., 2012) algorithm. Only the burnin by iteration combinations with the highest log probability are presented here. Values in bold italics represent the *K* assignment with the highest log probability.

| **Burnin** | **Iteration** | **Log Probability** | **K** |
| --- | --- | --- | --- |
| ***100*** | ***5*** | ***-9225.308*** | ***5*** |
| 200 | 6 | -9359.352 | 6 |
| 300 | 3 | -9373.429 | 6 |
| 400 | 5 | -9272.441 | 6 |
| 500 | 7 | -9358.917 | 5 |
